# Prognosis of palliative treatment for primary tracheal carcinoma: a two-center retrospective study

**DOI:** 10.3389/fonc.2025.1532005

**Published:** 2025-03-13

**Authors:** Qinyan Hong, Jun Teng, Yi Luo, Zhina Wang, Heng Zou, Lei Li, Nan Zhang, Hongwu Wang

**Affiliations:** ^1^ Respiratory Disease Center, Dongzhimen Hospital, Beijing University of Chinese Medicine, Beijing, China; ^2^ Graduate School, Beijing University of Chinese Medicine, Beijing, China; ^3^ Department of Oncology, Beijing Emergency General Hospital, Beijing, China; ^4^ Department of Pulmonary and Critical Care Medicine II, Beijing Emergency General Hospital, Beijing, China

**Keywords:** primary tracheal carcinoma, palliative treatment, initial tumor extension, long-term outcomes, prognostic analysis

## Abstract

**Introduction:**

More than half of patients with tracheal carcinoma (TC) do not receive radical treatment, but the clinical characteristics, palliative treatment options, and prognosis of this group remain unclear.

**Methods:**

This retrospective study analyzed 94 single primary TC patients (42 with tracheal squamous cell carcinoma [TSCC] and 52 with tracheal adenoid cystic carcinoma [TACC]) admitted to the Emergency General Hospital and Dongzhimen Hospital, Beijing University of Chinese Medicine. Kaplan-Meier survival curves, Log-rank tests, univariate and multivariate Cox and AFT models were used to assess overall survival (OS).

**Results:**

Among 89 patients without radical treatment, the median survival was 57 months, with 5-year and 10-year survival rates of 46.33% and 13.43%, respectively. Univariate analysis identified pathological type, smoking history, initial tumor extension (ITE), and targeted therapy as significant prognostic factors. The AFT model revealed that the median OS for TSCC patients was significantly shorter than for TACC patients, with a time ratio (TR) of 0.243 (95% CI: 0.153-0.386; *P* < 0.01), while targeted therapy was associated with a 1.790-fold increase in OS (TR: 1.790, 95% CI: 1.061-3.020; *P* = 0.029). Patients with extensive ITE had worse outcomes, with a TR of 0.628 (95% CI: 0.406-0.971; *P* = 0.037). Smokers had a TR of 0.601 (95% CI: 0.397-0.912; *P* = 0.017) compared with non-smokers. Subgroup analysis showed that smoking history was strongly associated with shorter OS in TSCC but not in TACC.

**Conclusions:**

Pathological type, ITE, targeted therapy and smoking history are important factors for evaluating the prognosis of TC patients receiving palliative treatment.

## 1 Introduction

Tracheal carcinoma(TC) is a rare malignancy with an annual incidence of 0.075 to 1 case per 100,000 individuals (1–3). Since 1980, clinical studies have demonstrated a substantial increase in the 5-year survival rate of TC, rising from 5.2% to 31.7% (3). The two primary pathological types, tracheal squamous cell carcinoma (TSCC) and tracheal adenoid cystic carcinoma (TACC), together account for more than 75% of cases (4). Most clinical studies on TC are based on data from large public databases, such as the Surveillance, Epidemiology, and End Results (SEER) program and the National Cancer Database (NCDB) (5, 6). Surgical resection is the preferred treatment for localized TC, and previous research has mainly focused on evaluating outcomes of surgery alone or in combination with other therapies (7, 8). However, due to the complex anatomical location and the high incidence of delayed or incorrect diagnosis, 60% to 80% of patients are not eligible for radical treatment (7, 9).

Palliative treatment is a crucial option for patients with TC who are not eligible for radical therapies (6, 10, 11). The main palliative treatment strategies for TC include radiotherapy, chemotherapy (platinum-based or other chemotherapeutic agents administered systemically or locally), targeted therapy (molecular targeted agents or vascular targeted agents such as Endostar delivered systemically or locally), immunotherapy, and bronchoscopic interventions, such as photodynamic therapy (PDT) and endobronchial stenting (12–15). Although palliative treatment has been extensively studied in various solid tumors due to its important role (16), research on its use in TC remains limited. This is primarily due to a lack of relevant variables in public databases and the predominance of single-center studies that focus on individual palliative approaches, leaving comprehensive evaluations of its overall effectiveness insufficiently explored (5, 9, 17–19).

This study retrospectively analyzed patients with single primary TC from two centers, detailing the clinical characteristics, evaluating palliative treatment options, and investigating potential factors influencing long-term prognosis.

## 2 Materials and methods

### 2.1 Study design and patients

This retrospective cohort study included 113 patients diagnosed with single primary TC at Beijing Emergency General Hospital (EG) and Dongzhimen Hospital, Beijing University of Chinese Medicine (DZM) between January 2010 and January 2023. Exclusion criteria were as follows: 1) prior radical treatment, including surgery or radiotherapy (radical surgery was defined as a procedure aiming for curative intent with complete resection of the tumor along with potentially involved surrounding tissues and lymph nodes, achieving negative surgical margins (6); radical radiotherapy was defined as radiation therapy with curative intent, delivered at an average dose exceeding 60 Gy (17)); and 2) incomplete tumor characteristic data. Ultimately, 94 patients who received palliative treatment were included in the final analysis (**Supplementary Figure S1**).

The study was conducted in accordance with the Declaration of Helsinki (2013 revision) and was approved by the ethics committees of DZM (No. 2024DZMEC-039-02) and EG (No. K24-24). Informed consent was waived by the ethics committees.

### 2.2 Data and definitions

Pathological subtype, age, sex, initial symptoms, smoking history, family history of cancer, and treatment history (including surgery, radiotherapy, chemotherapy, immunotherapy, targeted therapy, bronchoscopic interventional therapy, and PDT) were collected from medical records, interventional procedure reports, and pathological examination reports in the electronic medical record system.

Given the particular anatomical location of TC, we did not use the T stage in TNM staging but instead referred to the scheme proposed by Jin et al. to assess central airway stenosis (20).We believe this protocol offers a more suitable approach for the comprehensive evaluation of airway tumors in palliative treatment. Initial Tumor Extension(ITE) was categorized as I Zone, II Zone, and III Zone, based on invasion into the upper, middle, and lower thirds of the trachea. Initial Wall Invasion(IWI) was classified into 4 types: simply located in the lumen, outside the lumen, lumen wall, and mixed type. Initial Airway Narrowing(IAN) was defined based on the degree (%) of stenosis in the diameter of the trachea. Stenosis ≤25% was defined as Grade 1, 26%-50% as Grade 2, 51-75% as Grade 3, 76%-90% as Grade 4, and 91%-100% as Grade 5. We also collected data on regional lymph node metastasis and distant tumor metastasis. All patients underwent radiological and bronchoscopic examinations at the time of diagnosis. To ensure data consistency between the two participating centers, all original bronchoscopic images and CT scans were independently reviewed by two experienced radiologists and two experienced bronchoscopists. Any discrepancies were resolved through consensus.

The follow-up endpoint was overall survival (OS), defined as the time from pathological diagnosis to death from any cause or the last follow-up. Due to data limitations, cancer-specific mortality could not be distinguished. Survival data were obtained from the Chinese Center for Disease Control and Prevention or through telephone follow-up. The follow-up cutoff date was December 31, 2023. Patients with missing survival data were excluded from the survival analysis.

### 2.3 Statistical analysis

Quantitative data were expressed as the mean and standard deviation (SD). Significance levels were calculated using the equal variance t-test, Wilcoxon signed-rank test, or Mann-Whitney U test, depending on pathological grouping. Categorical data were presented as frequencies and percentages, and comparisons were made using the chi-square test or Fisher’s exact test.

Kaplan-Meier survival curves were constructed, and 5 -, 10 -, and 15-year survival rates, as well as median survival times, were calculated. The Log-rank test and univariate Cox regression analysis were initially performed to identify key prognostic variables. However, the proportional-hazards assumption was tested and found to be violated for pathological type and smoking history (**Supplementary Table S1**). Since multivariate Cox regression analysis relies on this assumption, it may not be suitable for our data. To address this limitation, we employed the accelerated failure time (AFT) model, which does not require the proportional-hazards assumption and allows for direct modeling of survival time. In the AFT model, covariate effects are expressed in terms of a Time Ratio (TR), where TR > 1 indicates prolonged survival time, while TR < 1 suggests a shortened survival time. The optimal distribution for the AFT model was determined based on the Akaike Information Criterion (AIC), with the model yielding the lowest AIC value selected for final analysis (21). In the subgroup analysis, univariate Cox regression analysis was initially performed by pathological group to identify variables with a significance level of *P* < 0.1. These selected variables were then included in a multivariate Cox regression model to adjust for other potential confounders, allowing for the identification of independent prognostic factors in each pathological subgroup.

All statistical analyses were performed using R software, version 4.4.0 (https://www.r-project.org/), and Rstudio 2024.04.1 + 748 (https://posit.co/). The following R packages were utilized: “arsenal”, “gtsummary”, “car”, “tidyverse”, “survival”, “ggplot2”, “dplyr”, “survival”, “survminer”, “readr”, “gridExtra”, “arsenal”, “forestplot”. All tests were two-sided, and *P* < 0.05 was considered statistically significant.

## 3 Results

### 3.1 Demographic and baseline characteristics

The demographic, baseline and therapeutic characteristics of the 94 patients are summarized in **Table 1**. TACC was the most common subtype(52/94, 55.3%), followed by TSCC (42/94, 44.7%). A total of 52.1% of the patients were former or current smokers. The most common initial symptoms were cough with expectoration (41.5%) and dyspnea (22.3%). Additionally, 69.1% of the patients had an ITE limited to one zone.

**Table 1:** Clinical features of the 94 patients with TC.

Characteristics	TACC, N = 52	TSCC, N = 42	*P* Value
Sex			0.003
Female	25 (48.1%)	8 (19.0%)	
Male	27 (51.9%)	34 (81.0%)	
Age (years)	47.0 ± 13.0	61.1 ± 12.5	<0.001
Initial Symptoms			<0.001
Cough & Sputum	27 (51.9%)	12 (28.6%)	
Dyspnea	16 (30.8%)	5 (11.9%)	
Hemoptysis	5 (9.6%)	18 (42.9%)	
Other Symptoms	4 (7.7%)	7 (16.7%)	
Smoking History			0.011
No	31 (59.6%)	14 (33.3%)	
Yes	21 (40.4%)	28 (66.7%)	
Family Cancer History			0.200
No	39 (75.0%)	36 (85.7%)	
Yes	13 (25.0%)	6 (14.3%)	
Initial Tumor Extension^a^			0.400
1	34 (65.4%)	31 (73.8%)	
2	13 (25.0%)	10 (23.8%)	
3	5 (9.6%)	1 (2.4%)	
Initial Airway Narrowing			0.800
1	6 (11.5%)	5 (11.9%)	
2	10 (19.2%)	10 (23.8%)	
3	17 (32.7%)	9 (21.4%)	
4	16 (30.8%)	16 (38.1%)	
5	3 (5.8%)	2 (4.8%)	
Initial Wall Invasion^b^			0.300
W1	17 (32.7%)	18 (42.9%)	
W2	35 (67.3%)	24 (57.1%)	
Tumor Metastasis			<0.001
Both	5 (9.6%)	1 (2.4%)	
Extra-pulm	2 (3.8%)	5 (11.9%)	
Intra-pulm	22 (42.3%)	3 (7.1%)	
None	23 (44.2%)	33 (78.6%)	
Lymph Node Status			0.089
No	35 (67.3%)	21 (50.0%)	
Yes	17 (32.7%)	21 (50.0%)	
Radiotherapy			0.110
No	20 (38.5%)	23 (54.8%)	
Yes	32 (61.5%)	19 (45.2%)	
Chemotherapy^c^			0.200
No	32 (61.5%)	20 (47.6%)	
Yes	20 (38.5%)	22 (52.4%)	
Immunotherapy^d^			0.130
No	48 (92.3%)	42 (100.0%)	
Yes	4 (7.7%)	0 (0.0%)	
Targeted Therapy^e^			0.140
No	38 (73.1%)	36 (85.7%)	
Yes	14 (26.9%)	6 (14.3%)	
PDT			0.071
No	45 (86.5%)	41 (97.6%)	
Yes	7 (13.5%)	1 (2.4%)	
Tracheoscopic Tx			0.600
Standard	30 (57.7%)	22 (52.4%)	
Standard&Stent	22 (42.3%)	20 (47.6%)	

^a^ ITE violation of any 1 of Zone I, Zone II, and Zone III was marked as 1, violation of 2 zones was marked as 2, and violation of 3 zones was marked as 3. When the invasion range was 1 Zone, it was defined as E1, and when the invasion range was more than 1 Zone, it was marked as E2. ^b^ In IWI, simply located in the lumen, outside the lumen, lumen wall, mixed type. Among the four types of invasion, the tumor with only one invasion type was defined as W1, and the tumor with no one invasion type was defined as W2. ^c^ Chemotherapy involved both bronchoscopic injections of gemcitabine and cisplatin, and systemic chemotherapy with platinum-based or other chemotherapeutic agents. ^d^ Immunotherapy: All four patients who received immunotherapy were diagnosed with TACC, and the immunotherapeutic agent used was IL-2. ^e^ Targeted therapy here refers to bronchoscopic administration of vascular-targeted drugs, including Endostar and Anlotinib.

Compared with TACC patients, TSCC patients were older(61.1 ± 12.5 vs 47.0 ± 13.0,*P <*0.001), had a higher proportion of males (81.0% vs 51.9%,*P* =0.003), and a greater percentage of smokers (66.7% vs 40.4%, *P* =0.011). The proportion of patients with tumor metastasis at the initial diagnosis was lower in TSCC than in TACC (21.4% vs 55.8%, *P <*0.001). The proportion of patients with lymph node metastasis in the initial diagnosis was higher in TSCC than in TACC, though the difference was not statistically significant (50.0% vs 32.7%, *P* =0.089).

### 3.2 Therapeutic characteristics

Compared with immunotherapy and targeted therapy, radiotherapy and chemotherapy were more commonly used as palliative treatments for TC patients, with 54.3% and 44.5% of patients undergoing radiotherapy and chemotherapy, respectively. A small number of patients received PDT, with the proportion of TSCC patients undergoing PDT being lower than that of TACC patients (2.4% vs 13.5%, *P* =0.071).

All patients received bronchoscopic interventions. Overall, we did not observe any statistically significant differences in the choice of treatment between TSCC and TACC patients. Due to the limited documentation of specific adverse reactions to various palliative treatments in the medical records, our study did not report on the adverse effects of palliative therapies.

### 3.3 Patient survival analysis

**Figure 1:**
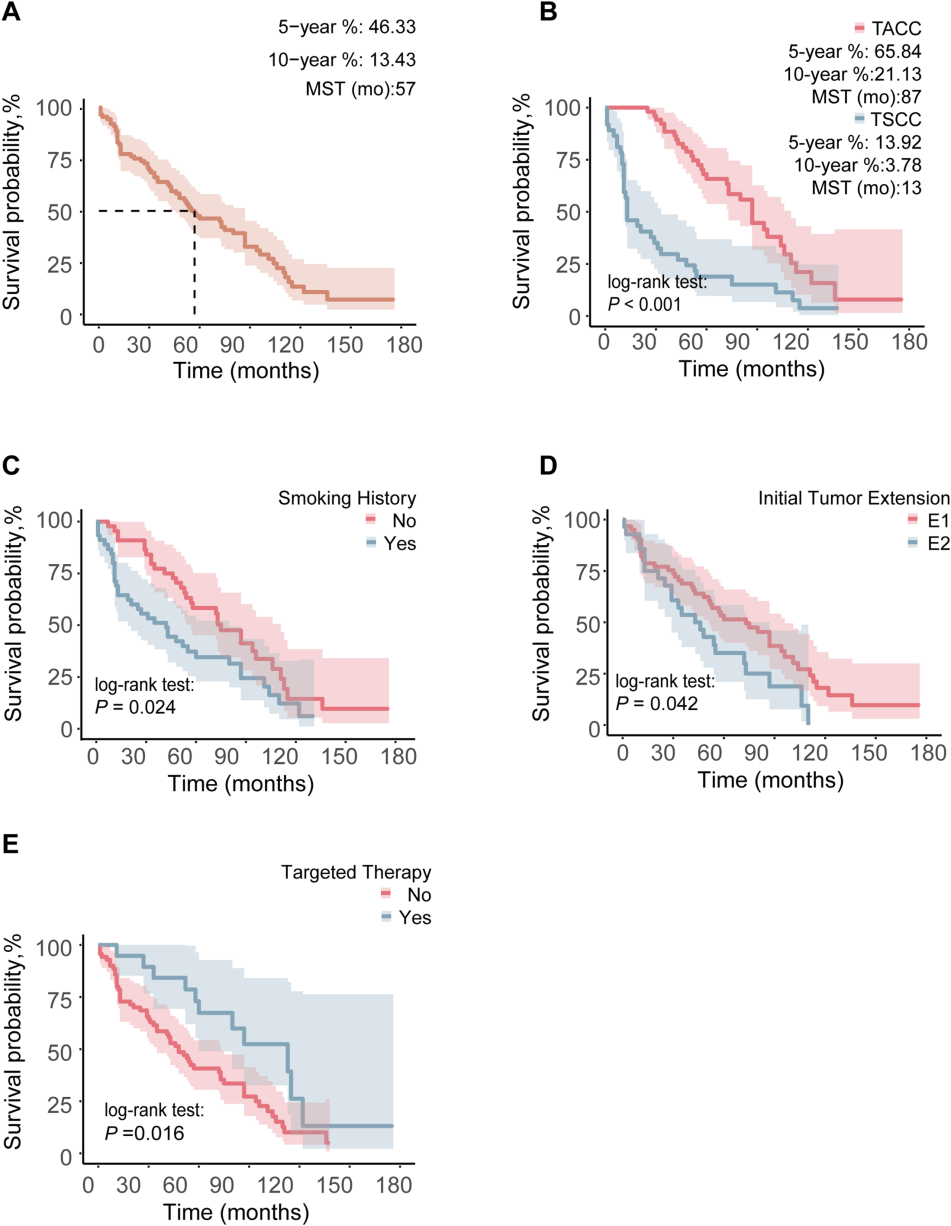
Comparison of OS between different groups in 89 TC patients: **(A)** OS curve of the whole population, **(B)** pathological type, **(C)** smoking history, **(D)** ITE, **(E)** targeted therapy. Any 1 of Zone I, Zone II, and Zone III invaded by ITE was marked as E1, and when the invasion area was more than 1 Zone, it was marked as E2.

We performed a survival analysis on 89 (37 TSCC and 52 TACC) TC patients with complete survival data, with a median follow-up duration of 53 months, during which 67 patients had died. The overall median survival time was 57 months, with TSCC patients having a median survival time of 13 months, compared to 87 months for TACC patients. The 5- and 10-year OS rates for the entire cohort were 13.4% and 46.3%, respectively. For TSCC, the 5- and 10-year survival rates were 13.9% and 3.8%, respectively, while for TACC, they were 65.8% and 21.1% (**Figures 1A, B**).

### 3.4 Kaplan-Meier and Cox regression analyses

Prognostic analysis was conducted on 89 patients. In the Kaplan-Meier analysis, factors significantly associated with longer OS included TACC (*P* < 0.001), absence of smoking history (*P* = 0.024), limited ITE (*P* = 0.042), targeted therapy (*P* = 0.016), and PDT (*P* = 0.043) (**Figures 1B–E**, **Supplementary Figure S2**).

Subsequently, univariate Cox regression analysis was performed for 89 patients (**Table 2**), revealing that TACC (*P* < 0.001), absence of smoking history (*P* = 0.025), limited ITE (*P* = 0.044), and targeted therapy (*P* = 0.018) were significantly associated with prolonged OS. We also conducted tests for the proportional-hazards assumption (**Supplementary Table S1**), which indicated that the proportional hazards assumption was violated for pathological type and smoking history. As a result, we opted not to proceed with multivariate Cox regression analysis.

**Table 2:** Univariate Cox regression analysis of OS in 89 TC patients.

Variable	HR (95%CI)	*P* Value
Pathology		<0.001
TACC	Reference	
TSCC	3.175 (1.950,5.168)	
Sex		0.061
Female	Reference	
Male	1.643 (0.978,2.759)	
Age	1.015 (0.997,1.034)	0.110
Symptoms		0.156
Hemoptysis	Reference	
Non-Hemoptysis	0.673 (0.390,1.163)	
Smoking History		0.025
No	Reference	
Yes	1.741 (1.071,2.831)	
Family Cancer History		0.857
No	Reference	
Yes	1.058 (0.571,1.962)	
Initial Tumor Extension^a^		0.044
E1	Reference	
E2	1.708 (1.014,2.876)	
Initial Airway Narrowing		0.603
I-III	Reference	
IV-V	0.875 (0.530,1.446)	
Initial Wall Invasion^b^		0.223
W1	Reference	
W2	1.372 (0.825,2.283)	
Tumor Metastasis	0	0.077
No	Reference	
Yes	0.641 (0.391,1.049)	
Lymph Node Status		0.859
No	Reference	
Yes	1.045 (0.640,1.707)	
Radiation		0.270
No	Reference	
Yes	0.759 (0.465,1.239)	
Chemotherapy		0.791
No	Reference	
Yes	1.067 (0.660,1.726)	
Immunotherapy		0.135
No	Reference	
Yes	0.408 (0.126,1.323)	
Targeted Therapy		0.018
No	Reference	
Yes	0.458 (0.239,0.877)	
PDT		0.055
No	Reference	
Yes	0.320 (0.100,1.027)	
Tracheoscopic Tx		0.132
Standard	Reference	
Standard & Stent	1.448 (0.894,2.346)	

^a^ ITE violation of any 1 of Zone I, Zone II, and Zone III was marked as 1, violation of 2 zones was marked as 2, and violation of 3 zones was marked as 3. When the invasion range was 1 Zone, it was defined as E1, and when the invasion range was more than 1 Zone, it was marked as E2. ^b^ In IWI, simply located in the lumen, outside the lumen, lumen wall, mixed type. Among the four types of invasion, the tumor with only one invasion type was defined as W1, and the tumor with no one invasion type was defined as W2.

Notably, in our cohort, targeted therapy referred to the bronchoscopic administration of vascular-targeting agents to facilitate tumor debulking (13, 15). Anlotinib and Endostar were the most commonly used agents, and no molecular targeted therapies were employed in this study.

### 3.5 AFT analysis

**Figure 2:**
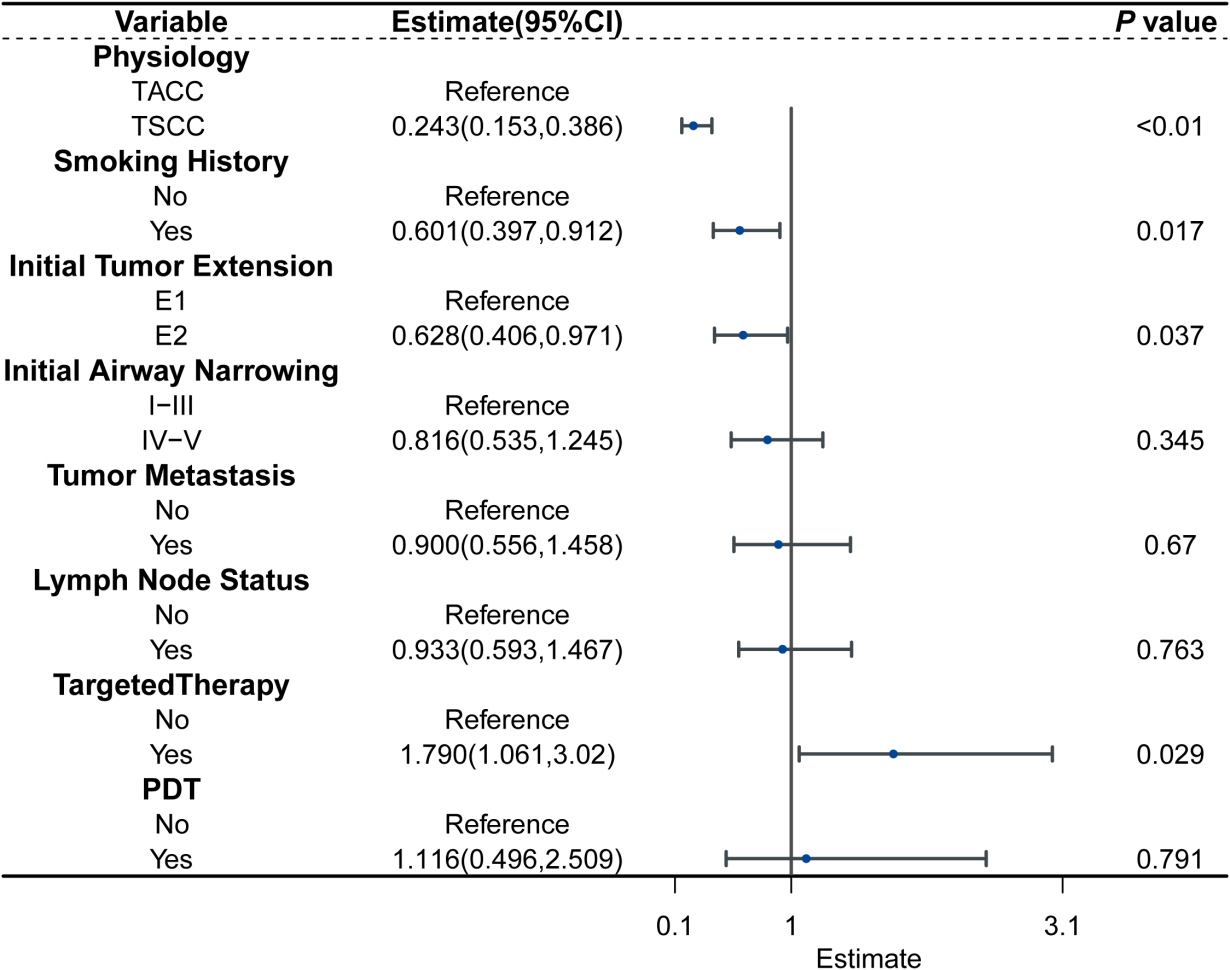
Forest plot for the AFT model characterizing the association between the variable and survival.

Covariates potentially related to survival were incorporated into the multivariate AFT analysis, based on clinical experience, Kaplan-Meier analysis, and univariate Cox regression results. The multivariate AFT analysis revealed that non-smoking (TR: 0.601, 95% CI:0.397-0.912; *P* = 0.017), TSCC(TR: 0.243, 95% CI:0.153-0.386; *P* < 0.01), limited ITE (TR: 0.628, 95% CI: 0.406-0.971; *P* = 0.037), and targeted therapy (TR: 1.790, 95% CI: 1.061-3.020; *P* = 0.029) were significantly associated with longer OS (**Figure 2**).

To ensure the robustness of the model, multicollinearity was assessed using the variance inflation factor (VIF). All covariates exhibited VIF values well below the commonly accepted threshold of 5, indicating that multicollinearity was not a concern in this analysis (**Supplementary Table S2**).

### 3.6 Subgroup analysis

**Figure 3:**
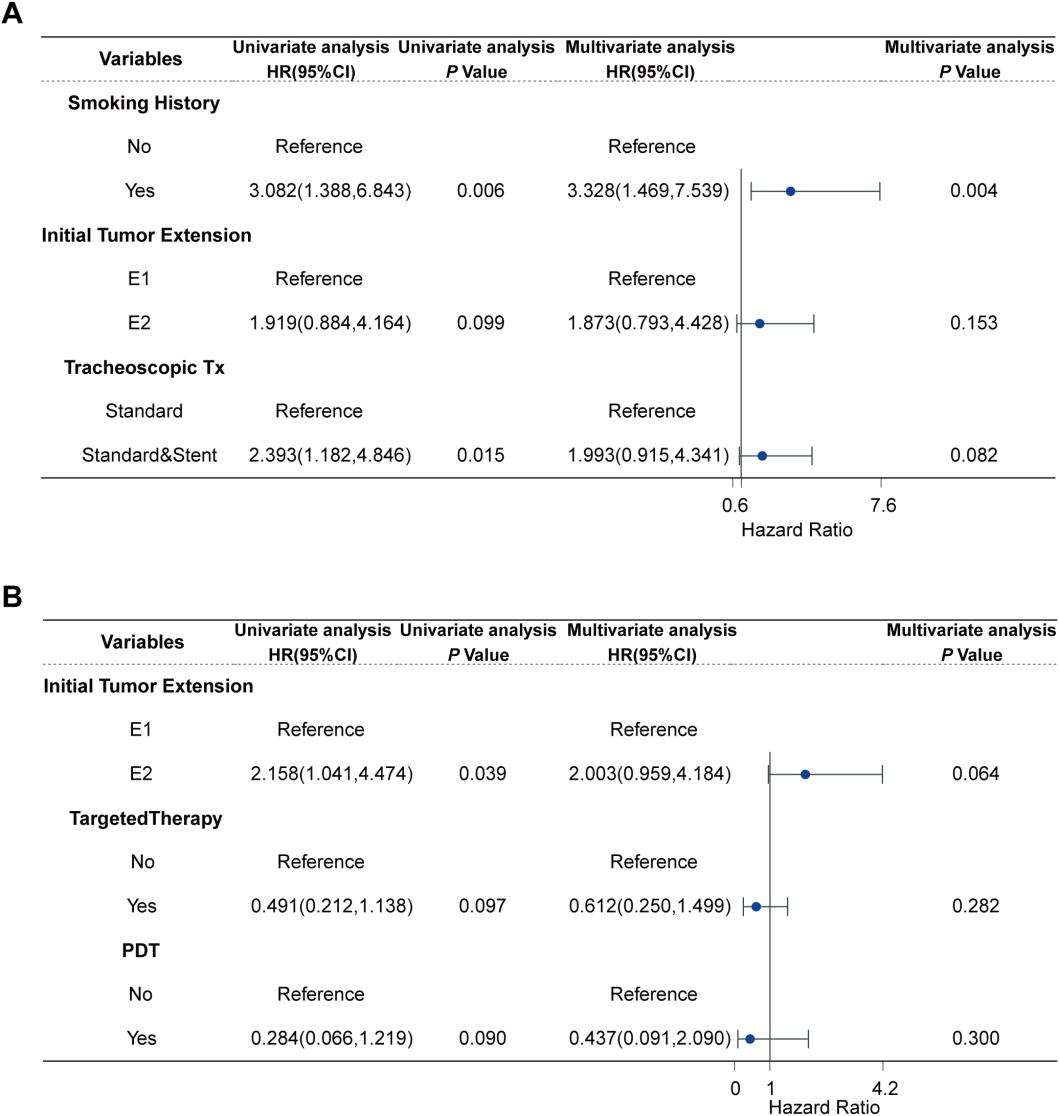
Cox regression analysis of TC patients: **(A)** Cox regression analysis of 37 TSCC, **(B)** Cox regression analysis of 52 TACC.

Subgroup analyses were conducted based on pathology. In the univariate Cox regression analysis of TSCC, non-smoking history (HR: 3.082, 95% CI:1.388-6.843; *P* =0.006), limited ITE(HR: 1.919, 95% CI: 0.884-4.164; *P* =0.099), and tracheoscopic debridement (HR: 2.393, 95% CI: 1.182-4.846; *P* =0.015) were significantly associated with longer OS. Variables with *a P* -value of less than 0.1 in the univariate Cox analysis were included in the multivariate analysis. In the multivariate Cox regression analysis of TSCC, smoking history (HR: 3.328, 95% CI: 1.469-7.539; *P* =0.004) was significantly associated with shorter OS. In the univariate Cox regression analysis of TACC, ITE (HR: 2.158, 95%CI:1.041-4.474; *P* = 0.039), targeted therapy (HR:0.612, 95%CI: 0.212-1.138; *P* = 0.097), and PDT(HR: 0.284, 95%CI:0.066-1.219; *P* = 0.090) were analyzed. The above variables were included in the multivariate analysis, but no variable in the multivariate Cox regression analysis for TACC had a statistically significant association with patient survival (**Figure 3**, **Supplementary Figures S3**, **S4**).

## 4 Discussion

This study enrolled 94 patients with TC who received palliative treatment at two centers. To our knowledge, this represents the largest two-center retrospective study on palliative treatment for TC, offering updated epidemiological insights into this condition. Furthermore, we analyzed prognostic factors for TC patients undergoing palliative treatment, identifying pathological type, smoking history, ITE, and targeted therapy as key determinants influencing the outcomes of palliative treatment.

Previous studies have indicated a gender disparity in the prevalence of TSCC, with a 2-4 times higher occurrence in males compared to females, while no such gender difference exists in TACC (12, 22). Our study identified a significant age difference in onset between TSCC and TACC, with the mean age of onset for TSCC being over 60 years, consistent with prior research showing a higher prevalence of TSCC in individuals over this age (12, 23). Consistent with previous findings, TSCC prevalence was strongly associated with smoking history, whereas TACC showed no significant correlation with smoking history (12, 23). Significant differences were also observed in the distribution of initial symptoms between TSCC and TACC; hemoptysis was frequently the first symptom in TSCC patients, potentially linked to ulcer formation caused by TSCC (12, 24). ITE was identified as an independent predictor of OS in TC, in line with prior studies that assessed ITE via CT or surgery (8). Although no significant differences were observed between TSCC and TACC in IAN and IWI, these parameters have introduced innovations in assessing tumors within the palliative treatment dimension and may hold potential for predicting recurrence or short-term prognosis in TC (25). Our study also revealed a statistically significant difference in tumor metastasis between TSCC and TACC, with TACC showing a higher rate of metastasis, contrasting with previous findings (12). However, no significant difference in lymph node metastasis was found between TSCC and TACC. Given that most patients in this study had lost the opportunity for radical treatment, resulting in a higher likelihood of lymphatic and distant metastasis, and some metastasis data were unknown, this result should be interpreted with caution (12, 26).

Previous studies have reported that only about 28% of TSCC patients undergo surgical treatment (6). One primary reason is the contraindications to airway surgery, including severe medical complications, extensive ITE, or a history of previous tracheal surgery, which may render patients ineligible for surgery. Additionally, the complexity of airway surgery means that only a limited number of medical institutions can perform these procedures (6, 27). Furthermore, the high risk of complications and mortality associated with surgery in TSCC patients further reduces the likelihood of surgical intervention (6). One study reported a 30-day mortality rate of 4.7% and a 90-day mortality rate of 10.5% in patients undergoing radical surgery (9). Postoperative complications, such as tracheoesophageal fistula, anastomotic dehiscence, airway stenosis, and recurrent laryngeal nerve injury, vary among institutions, with some studies reporting rates as high as 44.6% (6, 28). Most complications, except airway stenosis, typically occur within eight days post-surgery (29). Even at Massachusetts General Hospital, a leading institution in tracheal resection, the postoperative complication rate is 18.2% (27). Our study reports the survival outcomes of TSCC patients receiving palliative treatment, which, to our knowledge, has not been previously documented despite the substantial number of TSCC patients receiving such care. Palliative treatment has been widely employed in various solid tumors, particularly lung cancer (16, 30). Numerous studies have demonstrated that palliative treatment plays a crucial role in prolonging survival and alleviating symptoms in lung cancer patients (31–33). Importantly, key palliative treatments, such as radiotherapy and chemotherapy, carry relatively low risks (17). Common interventions like bronchoscopic interventions for TC also present lower rates of postoperative complications and are not limited by TC growth patterns (34, 35). It is noteworthy that although not statistically significant, stent placement appeared to be a potential risk factor for OS (*P* =0.082, **Figure 3A**). We believe that this is because patients requiring stent placement are usually those with severe tumor invasion, the disease itself may lead to poor prognosis, and stent placement can damage the airway, leading to complications such as airway stenosis above and below the stent and tracheoesophageal fistula (36). Nevertheless, the role of stent implantation should not be underestimated. In patients with severe tumor invasion, stents provide rapid and essential support for survival and significantly improve the quality of life (37). Other studies have also demonstrated a survival benefit associated with stenting (38).

The 5- and 10-year survival rates for TACC patients receiving palliative treatment were 65.84% and 21.13%, respectively, with a median survival time of 87 months. A recent study similarly reported 5- and 10-year survival rates of 63.7% and 46.4% in 28 TACC patients treated with non-surgical approaches (39). The discrepancy in 10-year survival rates between the studies may be attributed to the small sample size of 28 patients and the inclusion of patients receiving both radical radiotherapy and palliative treatment in the non-surgical cohort (39). Our study found that extensive ITE tended to be a risk factor for OS (*P*=0.060, **Figure 3B**), while other factors showed no significant impact on prognosis. TACC is a slow-growing, low-grade malignancy that often progresses very slowly, sometimes taking many years to worsen even without treatment. This indolent nature may partly explain why multivariate Cox regression did not show a significant palliative effect (12, 22, 39, 40). TACC is also characterized by a high recurrence rate, with positive surgical margins being a major risk factor (4, 39, 41). Previous studies have suggested that 59.8% of TACC surgery may have positive surgical margins, mainly because of the infiltrative growth characteristics of TACC that spreads in the submucosa and around the nerve (42). We do not deny that surgery remains the treatment of choice for localized TACC (12). However, considering the characteristics of easy recurrence and high metastasis, as well as the high difficulty and risk of operation, the applicability of surgical treatment has been reduced to a certain extent (43, 44). In this study, multiple palliative treatment regimens did not significantly prolong OS in TACC patients, which may be due to the indolent nature of TACC and the unclear palliative treatment status of some patients; therefore, these findings should be interpreted cautiously. The role of palliative treatment in TACC treatment has gained increasing recognition. A retrospective analysis by Lee et al. reported that none of the patients experienced disease progression within three months after receiving low-dose palliative radiotherapy (56.3–69.3 Gy) (18). The short-term efficacy of palliative treatment in TACC has also been demonstrated in previous cases (45). Recent research highlights the advantages of palliative treatment in preserving the trachea and delaying recurrence (46). Given its low risk, rapid symptom relief, and potential to maintain quality of life, palliative treatment remains the optimal choice when radical treatment is not feasible (11).

This study has several limitations. First, as a two-center retrospective study, the data were obtained from historical medical records, which may introduce selection and information biases. Second, due to the rarity of tracheal carcinoma, the study spanned a long period, potentially affecting the consistency of treatment strategies. Third, the retrospective nature of the data limited our ability to evaluate outcomes such as short-term prognosis, progression-free survival, quality of life, and psychological stress. In addition, we were unable to obtain data for mutation analysis. Despite these limitations, our findings provide valuable insights into the palliative treatment of TC. Future research should focus on defining the optimal timing, protocols, and duration of palliative interventions. Additionally, the individualization and complexity of palliative care regimens necessitate attention to treatment frequency, medication dosage, and the economic status of patients, as these factors may influence prognosis (9, 11).

## 5 Conclusions

This study summarizes the characteristics of palliative treatment for TC, highlighting that TACC, non-smoking history, limited invasion, and targeted therapy have a positive impact on prolonging OS in TC patients. The role of palliative treatment in TC needs to be further explored and verified, and more attention should be paid to non-clinical factors such as the initiation time, specific dosage, short-term prognosis, economic factors, and mental and emotional factors involved in palliative treatment programs to clarify the optimal treatment options for TC.

## Data Availability

The raw data supporting the conclusions of this article will be made available by the authors, without undue reservation.

## Supplementary material

Supplementary Figure S1 Flow diagram for the exclusion procedure of patients.

Supplementary Figure S2 Comparison of overall survival between different groups in 89 SPTC patients: **(A)** Sex, **(B)** Age, **(C)** Initial Symptoms, **(D)** Family Cancer History, **(E)** Initial Airway Narrowing, **(F)** Initial Wall Invasion, **(G)** Tumor Metastasis, **(H)** Lymph Node Status, **(I)** Radiation, **(J)** Chemotherapy, **(K)** Tracheoscopic Tx.

Supplementary Figure S3 Cox regression analysis of 37 SCC patients.

Supplementary Figure S4 Cox regression analysis of 52 ACC patients.
