# Supplementary figures and images for: Prognosis of palliative treatment for primary tracheal carcinoma: a two-center retrospective study
Source: Front Oncol. 2025 Mar 13;15:1532005. doi: 10.3389/fonc.2025.1532005 (PMC11966426; doi:10.3389/fonc.2025.1532005)

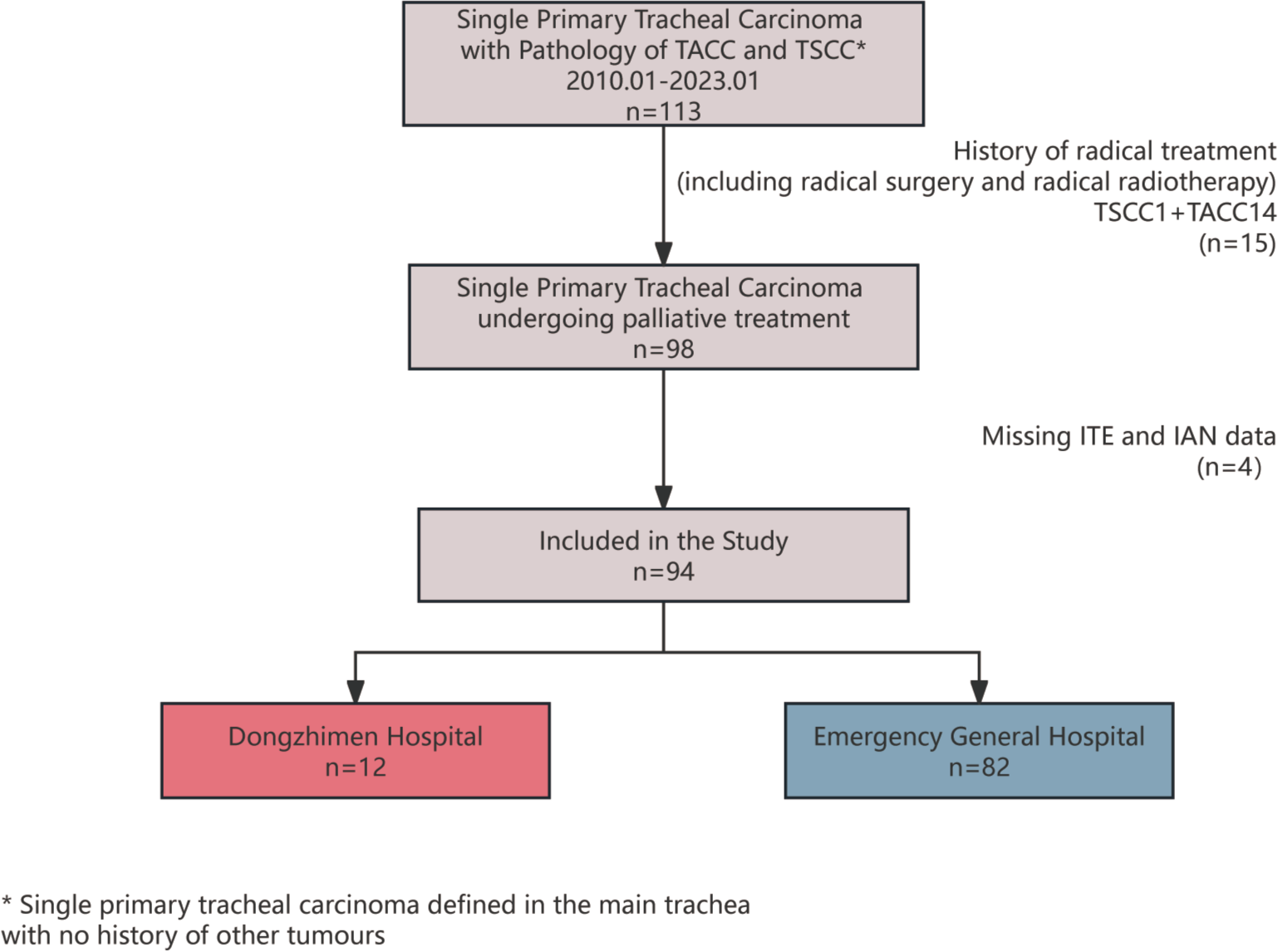

Supplement: Supplementary Figure S1 — Flow diagram for the exclusion procedure of patients. [file Image1.tif]

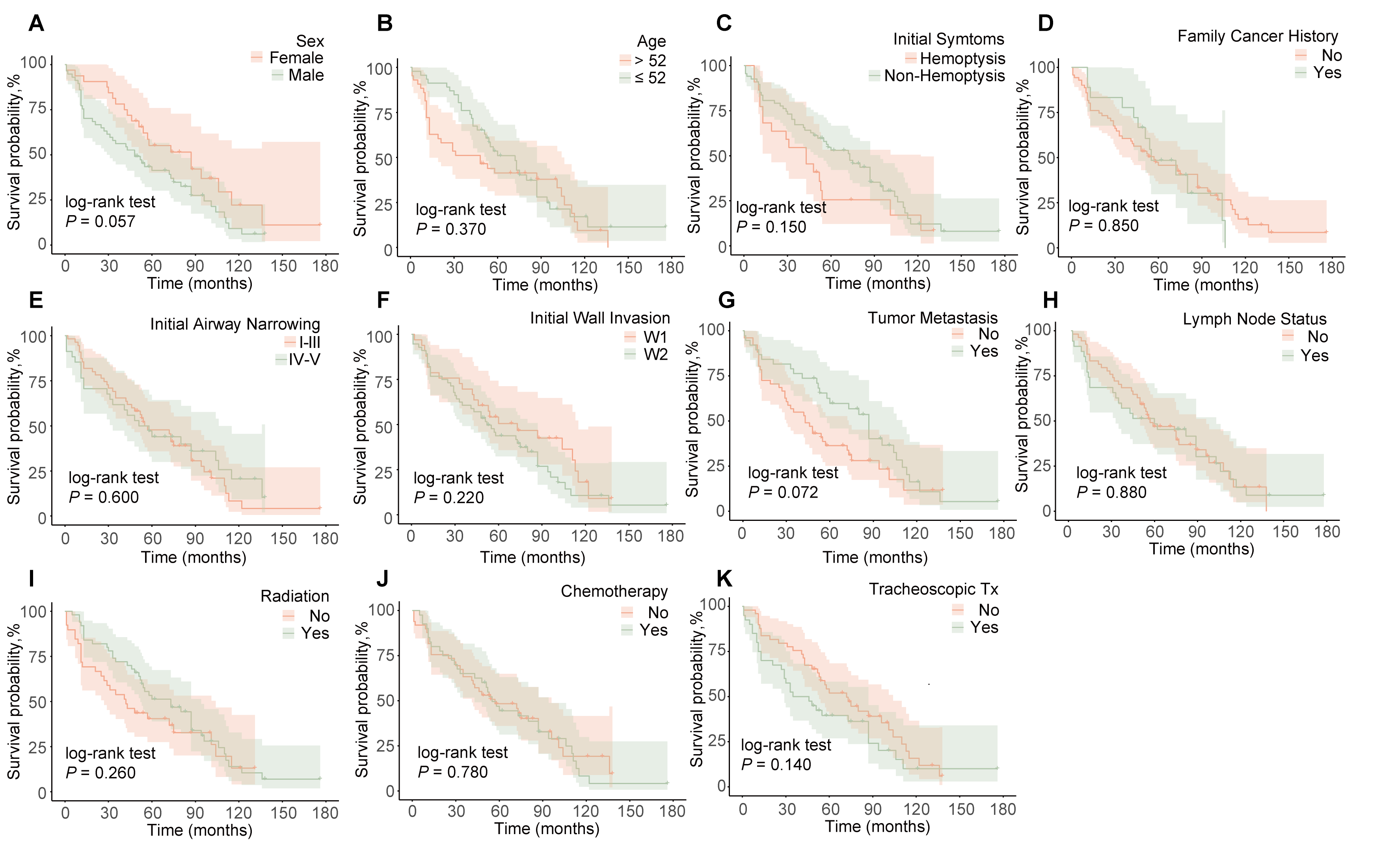

Supplement: Supplementary Figure S2 — Comparison of overall survival between different groups in 89 SPTC patients: (A) Sex, (B) Age, (C) Initial Symptoms, (D) Family Cancer History, (E) Initial Airway Narrowing, (F) Initial Wall Invasion, (G) Tumor Metastasis, (H) Lymph Node Status, (I) Radiation, (J) Chemotherapy, (K) Tracheoscopic Tx. [file Image2.tif]

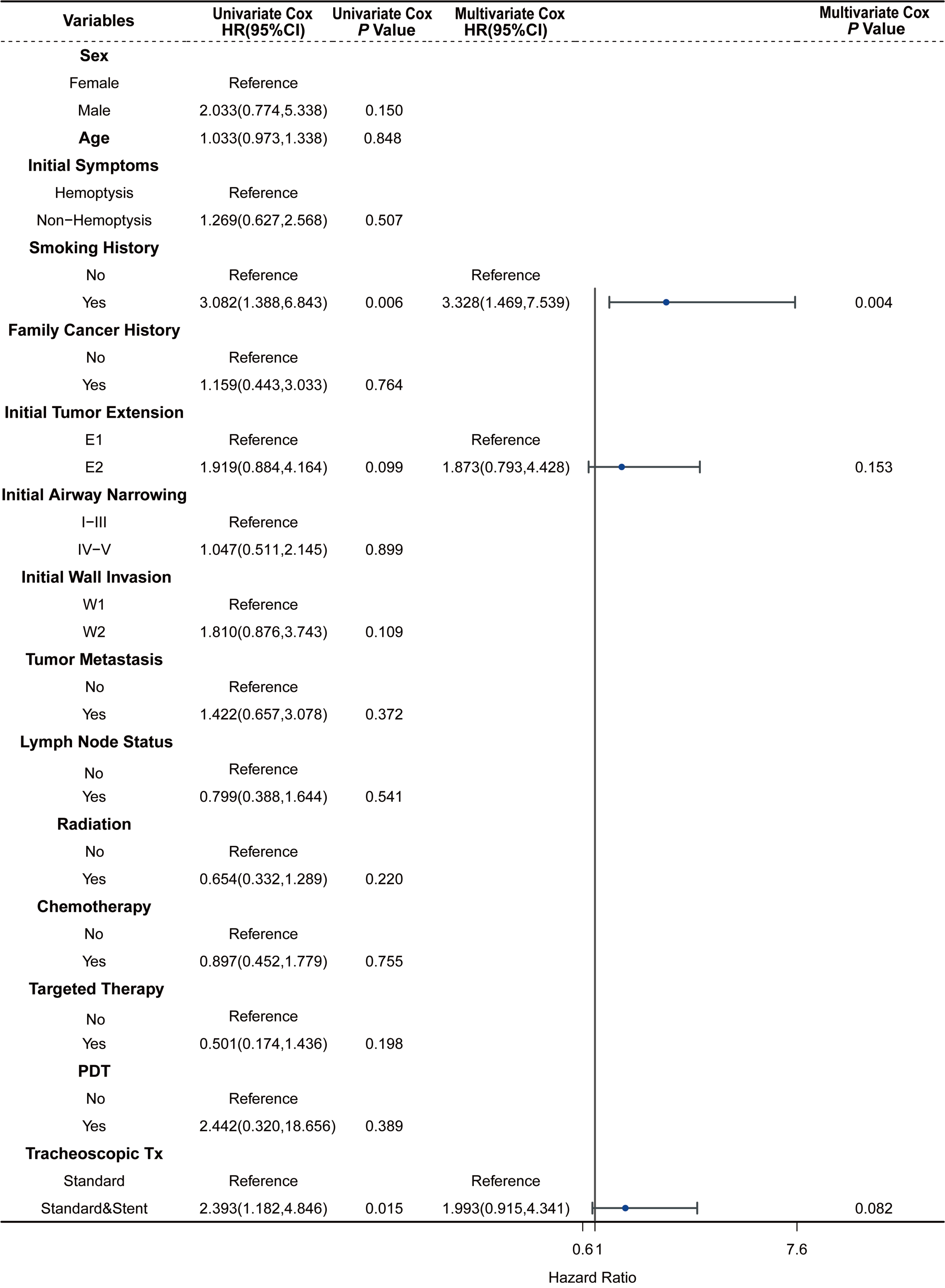

Supplement: Supplementary Figure S3 — Cox regression analysis of 37 SCC patients. [file Image3.tif]

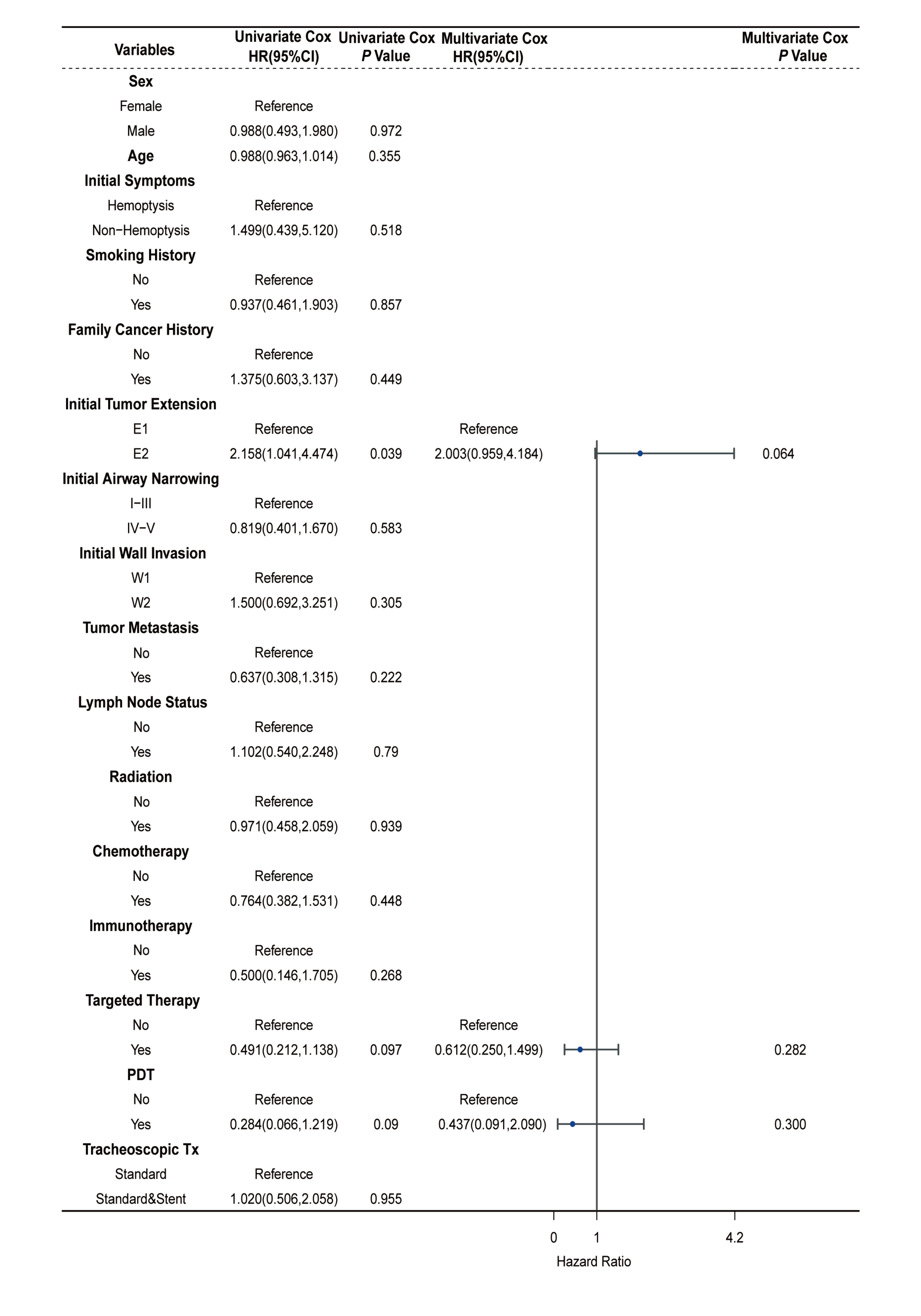

Supplement: Supplementary Figure S4 — Cox regression analysis of 52 ACC patients. [file Image4.tif]
