# Supplementary material for: Prognosis of palliative treatment for primary tracheal carcinoma: a two-center retrospective study
Source: Front Oncol. 2025 Mar 13;15:1532005. doi: 10.3389/fonc.2025.1532005 (PMC11966426; doi:10.3389/fonc.2025.1532005)
Supplement: Supplementary file 5 [file Table1.docx]

Table S1 Test of equal proportional hazards for univariate Cox

| Variable | rho | | Chi-square | *P* Value | |
| --- | --- | --- | --- | --- | --- |
| Physiology |  | |  |  | |
| ACC | Reference | | Reference |  | |
| SCC | -0.544 | | 17.364 | <0.001 | |
| Sex |  | |  |  | |
| Female | Reference | | Reference |  | |
| Male | -0.113 | | 0.986 | 0.321 | |
| Age | -0.211 | | 4.214 | 0.040 | |
| Initial Symptoms |  | |  |  | |
| Hemoptysis | Reference | | Reference |  | |
| Non- Hemoptysis | 0.114 | | 0.554 | 0.457 | |
| Smoking History |  | |  |  | |
| No | Reference | | Reference |  | |
| Yes | -0.246 | | 4.977 | 0.026 | |
| Family Cancer History |  | |  |  | |
| No | Reference | | Reference |  | |
| Yes | 0.221 | | 5.729 | 0.017 | |
| Initial Tumor Extension |  | |  |  | |
| E1 | Reference | | Reference |  | |
| E2 | 0.086 | | 0.901 | 0.343 | |
| Initial Airway Narrowing |  | |  |  | |
| I-III | Reference | | Reference |  | |
| IV-V | -0.218 | | 3.694 | 0.055 | |
| Initial Wall Invasion |  | |  |  | |
| W1 | Reference | | Reference |  | |
| W2 | 0.034 | | 0.039 | 0.844 | |
| Tumor Metastasis |  | |  |  | |
| No | Reference | | Reference |  | |
| Yes | 0.202 | | 1.940 | 0.164 | |
| Lymph Node Status |  | |  |  | |
| No | Reference | | Reference |  | |
| Yes | -0.140 | | 1.633 | 0.201 | |
| Radiation |  | |  |  | |
| No | Reference | | Reference |  | |
| Yes | 0.235 | | 4.562 | 0.033 | |
| Chemotherapy |  | |  |  | |
| No | Reference | | Reference |  | |
| Yes | 0.107 | | 1.043 | 0.307 | |
| Immunotherapy |  | |  |  | |
| No | Reference | | Reference |  | |
| Yes | 0.193 | | 1.785 | 0.181 | |
| Targeted Therapy |  | |  |  | |
| No | Reference | | Reference |  | |
| Yes | 0.166 | | 2.086 | 0.149 | |
| PDT |  | |  |  | |
| No | Reference | | Reference |  | |
| Yes | 0.016 | | 0.024 | 0.877 | |
| Tracheoscopic Tx |  |  | | |  |
| Standard | Reference | Reference | | |  |
| Standard & Stent | -0.175 | 2.594 | | | 0.107 |
