# Supplementary material for: Prognosis of palliative treatment for primary tracheal carcinoma: a two-center retrospective study
Source: Front Oncol. 2025 Mar 13;15:1532005. doi: 10.3389/fonc.2025.1532005 (PMC11966426; doi:10.3389/fonc.2025.1532005)
Supplement: Supplementary file 6 [file Table2.docx]

Table S2 Assessment of Multicollinearity in Covariates Included in AFT Analysis

| Variable | VIF |
| --- | --- |
| Initial Airway Narrowing | 1.041 |
| Pathology | 1.316 |
| Smoking History | 1.121 |
| Tumor Metastasis | 1.421 |
| Lymph Node Status | 1.188 |
| Initial Tumor Extension | 1.092 |
